# 

**Published:** 1884-12

**Authors:** 


					﻿vol.iv:
DECEMBER, 1884.
NO. 12.
THE
HOMEOPATHIC pHY^lClAfl,
A MONTHLY JOURNAL OF MEDICAL SCIENCE.
“ Seek the Truth: come whence it may, cost what it will.”
EDITED BY
EZDZMZTTIISTID J*. LEE, ILZE. D.,
CONTENTS:
(See inside of Cover.)
PHILADELPHIA :
No. 129 SOUTH THIRTEENTH STREET.
KEW ■jroitic::
A. L. CHATTERTON, PUBLISHING COMPANY, Publisher’s Agents.
LOKDOW:
ALFRED HEATH & CO., Sole Agents eor Great Britain,
114 Ebury Street, Eaton Square.
Yearly Subscription, $2.50, invariably in advance.
Entered at the Philadelphia Post-Office as Second-Class Mail Matter.
				

